# Common Genetic Variants of Surfactant Protein-D (SP-D) Are Associated with Type 2 Diabetes

**DOI:** 10.1371/journal.pone.0060468

**Published:** 2013-04-05

**Authors:** Neus Pueyo, Francisco J. Ortega, Josep M. Mercader, José M. Moreno-Navarrete, Monica Sabater, Sílvia Bonàs, Patricia Botas, Elías Delgado, Wifredo Ricart, María T. Martinez-Larrad, Manuel Serrano-Ríos, David Torrents, José M. Fernández-Real

**Affiliations:** 1 Service of Diabetes, Endocrinology and Nutrition (UDEN), Institut d’Investigació Biomédica de Girona (IdIBGi), CIBER de la Fisiopatología de la Obesidad y la Nutrición (CIBERobn, CB06/03/0010) and Instituto de Salud Carlos III (ISCIII), Girona, Spain; 2 Joint IRB-BSC Program on Computational Biology, Barcelona Supercomputing Center, Barcelona, Spain; 3 Institució Catalana de Recerca i Estudis Avançats (ICREA), Barcelona, Spain; 4 Hospital Central de Asturias, Oviedo, Spain; 5 Department of Internal Medicine II, Hospital Clínico San Carlos, CIBER de Diabetes y Enfermedades Metabólicas Asociadas (CIBERDEM), Madrid, Spain; University of Tor Vergata, Italy

## Abstract

**Context:**

Surfactant protein-D (SP-D) is a primordial component of the innate immune system intrinsically linked to metabolic pathways. We aimed to study the association of single nucleotide polymorphisms (SNPs) affecting *SP-D* with insulin resistance and type 2 diabetes (T2D).

**Research Design and Methods:**

We evaluated a common genetic variant located in the *SP-D* coding region (rs721917, Met^31^Thr) in a sample of T2D patients and non-diabetic controls (n = 2,711). In a subset of subjects (n = 1,062), this SNP was analyzed in association with circulating SP-D concentrations, insulin resistance, and T2D. This SNP and others were also screened in the publicly available Genome Wide Association (GWA) database of the Meta-Analyses of Glucose and Insulin-related traits Consortium (MAGIC).

**Results:**

We found the significant association of rs721917 with circulating SP-D, parameters of insulin resistance and T2D. Indeed, G carriers showed decreased circulating SP-D (p = 0.004), decreased fasting glucose (p = 0.0002), glycated hemoglobin (p = 0.0005), and 33% (p = 0.002) lower prevalence of T2D, estimated under a dominant model, especially among women. Interestingly, these differences remained significant after controlling for origin, age, gender, and circulating SP-D. Moreover, this SNP and others within the *SP-D* genomic region (i.e. rs10887344) were significantly associated with quantitative measures of glucose homeostasis, insulin sensitivity, and T2D, according to GWAS datasets from MAGIC.

**Conclusions:**

*SP-D* gene polymorphisms are associated with insulin resistance and T2D. These associations are independent of circulating SP-D concentrations.

## Introduction

Over nutrition and sedentary activities, in combination with repeated exposure to infectious agents and external injuries, could compromise the homeostasis of the innate immune system, leading to chronic subclinical inflammation, which is intrinsic to the metabolic syndrome [1]. Insulin resistance seems to be central to the pathophysiology of these alterations [2]. Approaches to this hypothesis have been made through the study of acute phase and innate immune proteins in association with insulin resistance and type 2 diabetes (T2D) [3]. Bactericidal/permeability-increasing protein, lipopolysaccharide binding protein, complement factors, α-defensins, and lactoferrin are examples of proteins from the innate immune system closely associated with metabolic parameters [4], [5], [6], [7].

Surfactant protein-D (SP-D) is a key-factor of the innate immunity system which develops its principal activity in lungs, protecting from inhaled microorganisms, organic antigens, and toxins [8]. SP-D modulates the leukocyte action contributing to the inflammatory response [9], and interacts with compounds such as bacterial lipopolysaccharides (LPS), oligosaccharides, and fatty acids [10]. The *SP-D* single nucleotide polymorphism (SNP) rs721917 (NC_000010.10: g.81706324A>G) is a *missense* substitution which leads the replacement in position 31 of an ancestral metionine by a threonine (Met^31^Thr) [10]. This polymorphic variation in the N-terminal domain of the SP-D molecule influences oligomerization, function, and circulating concentrations [11], leading to decreased immunologic capacity against bacteria [10]. Indeed, subjects carrying AA-genotype (Met/Met) have increased concentrations of SP-D in plasma [12], and show multimers, dodecamers, and monomers of subunits, whereas GG-carriers (Thr/Thr) produce almost exclusively monomers [11].

Previous evidence disclosed associations of low vital capacity and low weight at birth with an increased risk of T2D [13]. The close relationship between pulmonary acute phase proteins, the inflammatory state, and the development of metabolic complications has been also demonstrated [14], [15], [16], [17], [18]. However, the mechanisms of these interactions depend on scarcely known factors. In a previous manuscript, we provided data according to which low circulating SP-D concentrations were associated with increased fat accumulation and decreased insulin sensitivity [19]. Given the relationships among SP-D, insulin resistance and T2D, we hypothesized that genetic alteration in the former could be also associated with the prevalence of insulin resistance and T2D.

## Methods

### Subject Recruitment

Data and samples from 2,711 Caucasian subjects were obtained from population based prospective studies performed in three regions of Spain between 1996 and 1999∶680 subjects were recruited from the North-West (Asturias) [20], 1,341 from the center (Madrid) [21], and 690 from the North-East (Girona) [7]. Eligible participants were selected at random from the census and, after a screening visit, they were invited to participate. The participation rate was higher than 70%. The mean age of the participants was 51+/−12 years; 1,344 were men and 1,367 were women. Baseline studies included a standardized questionnaire, physical examination, and laboratory tests. Height and weight were measured with the participant in light clothing and without shoes by trained personnel using calibrated scales and a wall-mounted stadiometer, respectively. BMI was calculated by dividing weight in kilograms by the square of the height in meters. Waist circumference was measured to the nearest 0.5 cm midway between the lowest rib and the iliac crest using an anthropometric tape. Blood pressure was measured in the supine position on the right arm after a 10-min rest; a standard sphygmomanometer of appropriate cuff size was used and the first and fifth phases were recorded. Values used in the analysis are the average of three readings taken at 5 min intervals. Fasting serum and plasma was withdrawn and stored at −80°C until analysis. According to the American Diabetes Association Criteria a 75 g oral glucose tolerance test was performed in all subjects. Type 2 diabetes (T2D) was diagnosed in subjects having fasting plasma glucose >7 mM and two-hour post-load plasma glucose >11.1 mM after the oral glucose tolerance test. T2D patients were also prospectively recruited from outpatient clinics on the basis of a stable metabolic control in the previous 6 months, as defined by stable HbA1c values. Inclusion criteria were: 1) absence of systemic and metabolic disease other than obesity and T2D, and 2) absence of infection within the previous month. Liver disease and thyroid dysfunction were specifically excluded by biochemical work-up. Other exclusion criteria included the following: 1) clinically significant hepatic, neurological, endocrinologic, or other major systemic disease, including malignancy; 2) history or current clinical evidence of hemochromatosis; 3) history of drug or alcohol abuse, defined as >80 g/day in men and >40 g/day in women; 4) elevated serum creatinine concentration; 5) acute major cardiovascular event in the previous 6 months; 6) acute illnesses and current evidence of acute or chronic inflammatory or infective diseases; and 7) mental illness rendering the subjects unable to understand the nature, scope, and possible consequences of the study. All participants were requested to withhold alcohol and caffeine during at least 12 h prior to the different tests. All subjects gave written informed consent after the purpose of the study was explained to them. The experimental protocol was approved by the Ethics Committee of all participant institutions, including the *Hospital Central de Asturias* (Asturias, Spain), the *Hospital Clínico San Carlos* (Madrid, Spain), and the *Hospital Universitari de Girona Dr. Josep Trueta* (Girona, Spain), respectively, so we certify that all applicable institutional regulations concerning the ethical use of information and samples from human volunteers were followed during this research.

### SP-D Gene Polymorphisms

The *SP-D* gene polymorphism rs721917 (NC_000010.10:g.81706324A>G) was analyzed using iPLEX™ chemistry on a MALDI-TOF Mass Spectrometer (*Sequenom Inc., San Diego, CA, USA*), which is a multiple assay format. This SNP was mixed with other SNPs corresponding to different genes that are under study in our laboratories. The remaining procedure was similar to a previously described method for PCR reactions [22]. The products were spotted on a SpectroChip (Sequenom Inc.), processed and analysed in a Compact Mass Spectrometer by Mass-ARRAY Workstation (version 3.3) software (*Sequenom Inc.*). The high-throughput genotyping assays were performed at the genotyping facilities of *Centro Nacional de Genotipado* (CeGen), in the Node of Santiago.

The intergenic gene polymorphism rs10887344 (NC_000010.10:g.81768162G >A) was genotyped in the subpopulation of subjects recruited at the North-West of Spain (Asturias) by means of allelic discrimination assays using an ABI Prism 7000 sequence detector and TaqMan technology (Applied Biosystems, Foster City, CA, USA). The reaction was performed in a final volume of 25 µl. DNA was amplified after 50 cycles with an initial denaturation of 10 min at 95°C. The cycle program consisted of 15 sec denaturation at 92°C and 1 min annealing and extension at 60°C. Positive and negative controls, which were correctly identified, were included in all reactions.

### Analytical Determinations

Commercially available (BioVendor GmbH, Heidelberg, Germany) solid-phase enzyme-linked immunosorbent assays (ELISA) based on the sandwich principle were used for the in vitro quantitative determination of human surfactant protein-D (n = 1,065) concentrations in plasma. Intra- and inter- assay coefficients of variation for these determinations were between 5–10%. Other biochemical measurements were performed as previously described [6].

### Statistical Analyses

The statistical association between the *SP-D* gene polymorphism rs721917 and type 2 diabetes (T2D) was assessed using custom-written software based on R environment [23]. Departures from Hardy-Weinberg equilibrium were tested in controls using a chi-square goodness of fit test with one degree of freedom. The risk of developing T2D under exposure to this *SP-D* SNP was evaluated using logistic regression to estimate Odd Ratios (OR), considering the AA genotype as the reference group. Hereby, codominant and dominant (fitting a dominant model for AA-genotype carriers) models that included the origin of the samples, age, and sex effects was fitted to estimate the ORs between the exposure to the GG, AG, and AA genotype. An additional model (Log-additive) was also fitted under these conditions. In a second assay of significance, circulating SP-D concentrations were also included as covariate. Other statistical analyses and graphics were performed using the program SPSS (IBM SPSS Statistics, Chicago, IL, USA). One-way ANOVA for multiple comparisons, using post-hoc by Bonferroni’s test and the test t-Student when equal variances could be assumed, was used to compare groups with respect to continuous variables.

In order to replicate the associations found in our population, we evaluated the *SP-D* gene polymorphism rs721917 (NC_000010.10: g.81706324A>G) and screened for other associations within the *SP-D* genome region in data from publicly available datasets from the Meta-Analyses of Glucose and Insulin-related traits Consortium (MAGIC). Seven glycemic traits were evaluated in genome wide association (GWA) databases of MAGIC, which includes data on continuous glycaemic traits in a sample of T2D patients and non-diabetic controls [24], [25], [26], [27], [28]. Standard Chi-Squared Tests and logistic regression tests were used to estimate ORs.

## Results

### Gene Association Results

**Figure 1:**
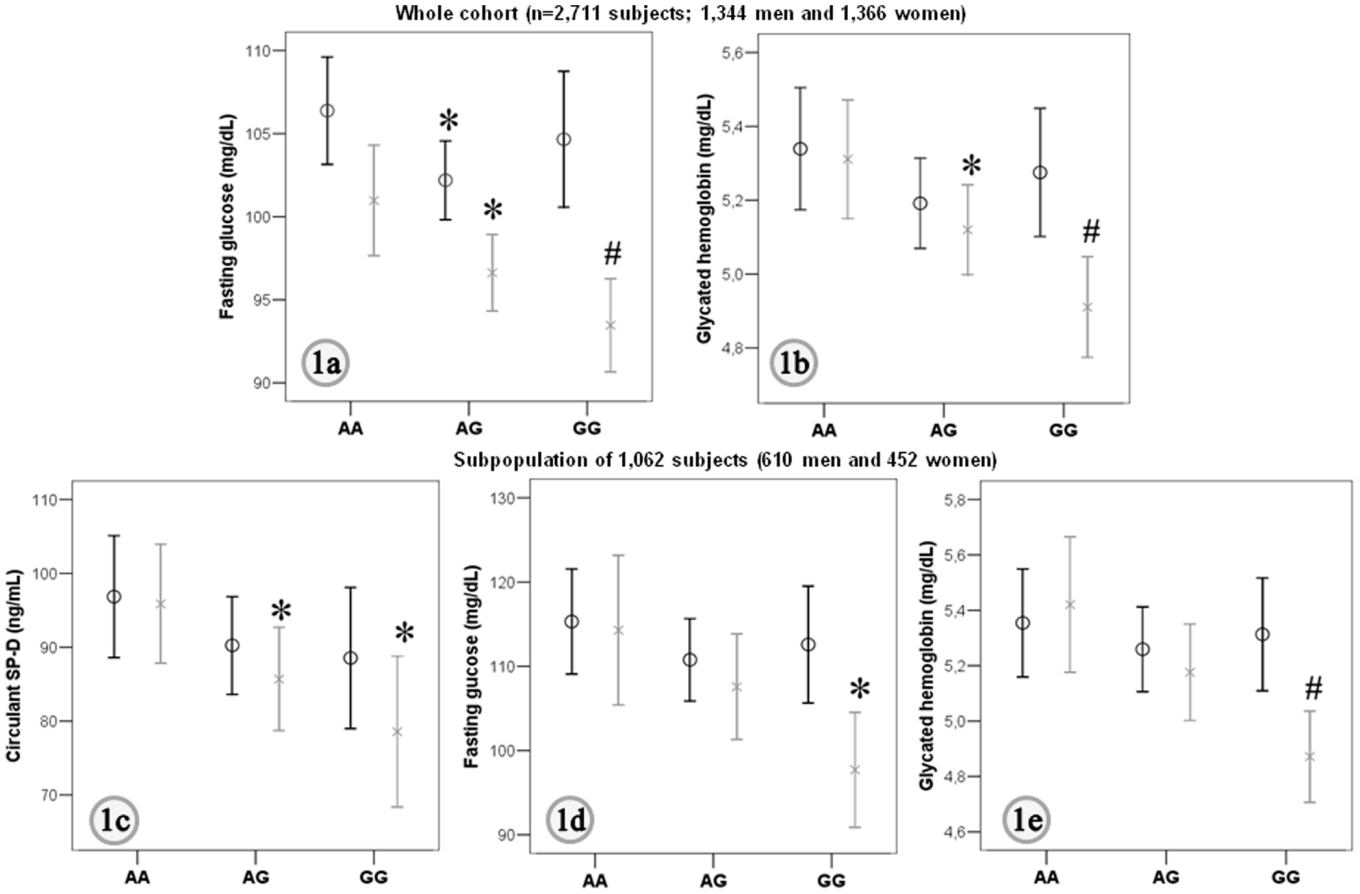
Genotypes for rs721917 and measures of impaired glucose tolerance and circulating SP-D. Mean and 95% confidence interval for fasting glucose (**Fig. 1a**) and glycated hemoglobin (**Fig. 1b**) for the whole cohort (n = 2,711), and for circulating SP-D concentration (**Fig. 1c**), fasting glucose (**Fig. 1d**) and glycated hemoglobin (**Fig. 1d**) in a subpopulation of subjects (n = 1,062) according to the genotypes for rs721917. Men: black bars, women: grey bars. *p<0.05 and #p<0.0001 for comparisons with AA-genotype.

The frequency of the G allele in the chromosome 10 at the position rs721917 NC_000010.10: g.81706324A>G (Contig position) of the *surfactant protein-D* (*SP-D*) gene was lower in subjects with impaired glucose tolerance and T2D (IGT, ***Table 1***). Indeed, the increased prevalence of the A allele run in parallel to increased circulating SP-D concentrations (***Table 1***), fasting glucose, and glycated hemoglobin in women, but not in men (***Figure 1a and 1b***).

**Table 1:** Subjects’ characteristics according to the single nucleotide polymorphism rs721917 (NC_000010.10:g.81706324A>G, Met^31^Thr) for *SP-D* gene.

Genotype	AA	AG	GG	p†	p*	p**
**Subjects (M)**	**468**	**664**	**212**			
**Smokers (%)**	37	33	36			
**T2D (%)**	**14**	**13**	**16**			
**Age (years)**	51±12	52±12	53±12	0.431	0.275	0.215
**BMI (kg/m^2^)**	27.9±4.3	27.8±4.3	28.3±4.5	0.27	0.974	0.238
**WHR**	0.95±0.07	0.96±0.08	0.95±0.06	0.111	0.158	0.868
**Fasting Glucose** **(mg/dL)**	**106.5±35.5**	**102.1±31.1**	**104.3±29.8**	0.083	**0.048**	0.438
**Glucose 2** **h post-overload (mg/dL)**	**108.5±39.4**	**110.8±46.7**	**121.6±49.4**	**0.006**	0.076	**0.003**
**Fasting Insulin (mU/L)**	**11.2±9**	**10.2±6.4**	**12.2±10.8**	**0.011**	0.353	0.225
**Insulin 2** **h post-overload (mU/L)**	**50.2±55.5**	**43.3±49.5**	**66±70.3**	**0.003**	0.894	**0.036**
**HOMA_IR_**	**2.74±2.26**	**2.49±1.69**	**3.04±3.03**	**0.008**	0.383	0.186
**HbA_1c_ (%)**	5.3±1.4	5.2±1.2	5.3±1.1	0.363	0.217	0.562
**SP-D (ng/mL)** #	98.9±68.3	92.5±67.8	91.5±62.4	0.498	0.24	0.336
**Subjects (F)**	**501**	**641**	**225**			
**Smokers (%)**	17	17	17			
**T2D (%)**	**13**	**10**	**8**			
**Age (years)**	52±12	51±12	52±12	0.478	0.415	0.942
**BMI (kg/m^2^)**	29.5±7.3	28.8±6.4	29±6.2	0.189	0.073	0.339
**WHR**	0.84±0.07	0.84±0.07	0.85±0.08	0.627	0.542	0.344
**Fasting Glucose** **(mg/dL)**	**100.9±37.8**	**96.6±29.6**	**93.5±21.4**	**0.007**	**0.008**	**0.001**
**Glucose 2** **h post-overload (mg/dL)**	**108.6±38.9**	**102±31.7**	**103.7±32.5**	**0.023**	**0.011**	0.152
**Fasting Insulin (mU/L)**	11.1±12	10.4±6.4	10.5±6.3	0.606	0.32	0.593
**Insulin 2** **h post-overload (mU/L)**	37.2±35.2	35.4±35.7	32.2±42.5	0.67	0.523	0.384
**HOMA_IR_**	2.58±2.71	2.38±1.59	2.39±1.56	0.311	0.187	0.374
**HbA_1c_ (%)**	**5.3±1.3**	**5.1±1.1**	**4.9±0.7**	**0.006**	**0.01**	**<0.0001**
**SP-D (ng/mL)** #	**95.9±52.8**	**85.7±51.2**	**78.6±44.4**	**0.03**	**0.015**	**0.009**

**T2D:** Type 2 diabetes; **BMI:** Body mass index; **WHR:** Waist-to-hip ratio; **HbA_1c_:** Glycated hemoglobin; **HOMA_IR_:** Homeostasis model assessment of insulin resistance; **SP-D:** Surfactant protein-D.

# These determinations were performed in a subpopulation of 1,062 subjects.

† for the comparison by ANOVA among the different genotypes;

* for the comparison by test *t*-student between AA and non-AA individuals, and

** between AA and GG carriers in the whole cohort. Significant differences are shown in **bold**.

The study had a power >75% for estimating ORs >1.7 with a MAFs >0.70 and α = 0.05. The similar ORs for the AG and GG genotypes obtained for the codominant model suggest the possibility of fitting a dominant model for AA-genotype carriers (***Table 2***). In this case, the residual deviance of the genotype, once origin, age, and gender were added to the model, reached a p-value <0.001. An additional model (Log-additive) that included origin, age, and gender effects was also fitted. GG homozygotes had 40% (p = 0.0025) lower prevalence of T2D than A-allele carriers, estimated by the Log-additive model (***Table 2***). In agreement, significantly decreased fasting glucose (p = 0.0003) and glycated hemoglobin (p = 0.0006) was found in the whole cohort of G allele carriers (***Table 2***). No significant differences between genotypes were found for fasting insulin in this population. Similar results were found in additional models that included the effect of BMI or other anthropometrical parameters. Some T2D participants were under treatment (fibrates, statins, oral hypoglycemic drugs, and/or insulin). However, it should be noted that the main findings remained essentially unchanged after the exclusion of these subjects (data not shown).

**Table 2:** Codominant, dominant, and Log-additive models for *SP-D* gene polymorphism rs721917 (NC_000010.10:g.81706324A>G, Met^31^Thr) in the whole cohort after correcting by origin of the sample, age, and sex-effects.

	No Type 2 Diabetes		Type 2 Diabetes				
Model	N	%	N	%	OR	OR CI (95%)	P (> |z|)
**Codominant**							
A/A	790	34.8	178	40.5	**1**		**0.009**
A/G	1102	48.5	203	46.1	**0.82**	**[0.65/1.04]**	
G/G	378	16.7	59	13.4	**0.6**	**[0.43/0.84]**	
**Dominant**							
A/A	790	34.8	178	40.5	**1**		**0.016**
A/G-G/G	1480	65.2	262	59.5	**0.76**	**[0.61/0.95]**	
**Log-Additive**							
(1, 2, 3)	2270	83.8	440	16.2	**0.79**	**[0.67/0.92]**	**0.002**
	**Fasting Glucose (mg/dL)**						
**Model**	**N**	**Median**	**SE**	**Variation**		**CI (95%)**	**P (> |z|)**
**Codominant**							
A/A	967	103.6	1.18				**0.001**
A/G	1305	99.5	0.84	**−3.57**		**[−6.1/−1.1]**	
G/G	437	98.9	1.27	**−5.8**		**[−9.2/−2.4]**	
**Dominant**							
A/A	967	103.6	1.18				**<0.001**
A/G-G/G	1742	99.3	0.71	**−4.13**		**[−6.4/−1.8]**	
**Log-Additive**							
(1, 2, 3)	**−**3.03	**−**4.66	**−**1.41				**<0.001**
	**HbA1c (mg/dL)**						
**Model**	**N**	**Median**	**SE**	**Variation**		**CI (95%)**	**P (> |z|)**
**Codominant**							
A/A	537	5.33	0.059				**0.003**
A/G	710	5.16	0.044	**−0.15**		**[−0.27/−0.03]**	
G/G	256	5.11	0.058	**−0.27**		**[−0.44/−0.11]**	
**Dominant**							
A/A	537	5.33	0.058				**0.002**
A/G-G/G	966	5.15	0.036	**−0.18**		**[−0.30/−0.07]**	
**Log-Additive**							
(1, 2, 3)	**−**0.14	**−**0.22	**−**0.06				**<0.001**
	**SP-D (ng/mL)**						
**Model**	**N**	**Median**	**SE**	**Variation**		**CI (95%)**	**P (> |z|)**
**Codominant**							
A/A	377	96.4	2.95				**0.014**
A/G	494	88.3	2.45	**−8.18**		**[−15.3/−1.1]**	
G/G	191	84.6	3.57	**−12.48**		**[−21.7/−3.2]**	
**Dominant**							
A/A	377	96.4	2.95				**0.005**
A/G-G/G	685	87.3	2.03	**−9.38**		**[−16.1/−2.7]**	
**Log-Additive**							
(1, 2, 3)	**−**6.65	**−**11.05	**−**2.06				**0.004**

**OR** are odd ratios and **OR CI (95%)** are their respective 95% confidence interval (CI). P (> |z|) is the P-value of the z-test that tests if the regression coefficients of the model can be assumed to be zero. Significant differences are shown in **bold**.

### Circulating SP-D Concentrations

To further study the possible biological significance of this association we hypothesized a link among the *SP-D* gene polymorphism rs721917, circulating SP-D concentrations, and risk factors for T2D. In this sample of subjects (n = 1,062), G-allele carriers had decreased circulating SP-D than AA-subjects (p-additive = 0.0042, *Table 1*). Currently, the increased frequency of the A allele run in parallel to increased sex-adjusted SP-D concentrations (*Fig. 1c*), fasting glucose (p-additive = 0.004, *Fig. 1d*), and glycated hemoglobin (p-additive = 0.0035, *Fig. 1e*), especially among women. Of note, the significance of differences in fasting glucose between groups after controlling for origin, age, and gender remained significant when correcting for SP-D concentrations in plasma (*Table 3*).

**Table 3:** Codominant, dominant, and Log-additive models for *SP-D* gene polymorphism rs721917 (NC_000010.10:g.81706324A>G, Met^31^Thr) after correcting by origin of the sample, age, sex, and concentrations of SP-D in plasma.

	No Type 2 Diabetes		Type 2 Diabetes				
Model	N	%	N	%	OR	OR CI (95%)	P (> |z|)
**Codominant**							
A/A	272	34.1	105	39.6	**1**		**0.039**
A/G	373	46.8	121	45.7	**0.81**	**[0.58/1.13]**	
G/G	152	19.1	39	14.7	**0.57**	**[0.36/0.89]**	
**Dominant**							
A/A	272	34.1	105	39.6	1		0.051
A/G-G/G	525	65.9	160	60.4	0.73	[0.54/1.00]	
**Log-Additive**							
(1, 2, 3)	797	75	265	25	**0.76**	**[0.62/0.94]**	**0.012**
	**Fasting Glucose (mg/dL)**						
**Model**	**N**	**Median**	**SE**	**Variation**		**CI (95%)**	**P (> |z|)**
**Codominant**							
A/A	377	114.9	2.6				**0.016**
A/G	494	109.4	1.97	**−5.61**		**[−11.3/0.1]**	
G/G	191	106.7	2.57	**−10.42**		**[−17.8/−3.0]**	
**Dominant**							
A/A	377	114.9	2.66				**0.011**
A/G-G/G	685	108.7	1.59	**−6.94**		**[−12.3/−1.6]**	
**Log-Additive**							
(1, 2, 3)	−5.28	−8.88	−1.68				**0.004**
	**HbA1c (mg/dL)**						
**Model**	**N**	**Median**	**SE**	**Variation**		**CI (95%)**	**P (> |z|)**
**Codominant**							
A/A	372	5.38	0.078				**0.014**
A/G	488	5.22	0.058	**−0.15**		**[−0.31/0.01]**	
G/G	189	5.14	0.072	**−0.302**		**[−0.51/−0.09]**	
**Dominant**							
A/A	372	5.38	0.078				**0.012**
A/G-G/G	677	5.2	0.047	**−0.192**		**[−0.34/−0.04]**	
**Log-Additive**							
(1, 2, 3)	−0.15	−0.25	−0.05				**0.003**

**OR** are odd ratios and **OR CI (95%)** are their respective 95% confidence interval (CI). P (> |z|) is the P-value of the z-test that tests if the regression coefficients of the model can be assumed to be zero. Significant differences are shown in **bold**.

On the other hand, circulating SP-D concentrations were significantly decreased in T2D when compared to non-diabetic subjects in both non-obese and obese individuals, and inversely associated with BMI, fat mass, fasting glucose, and fasting triglycerides, as previously reported [19], and independently of genetic variations.

### Validation of the Association through Screening of MAGIC GWA Databases

**Figure 2:**
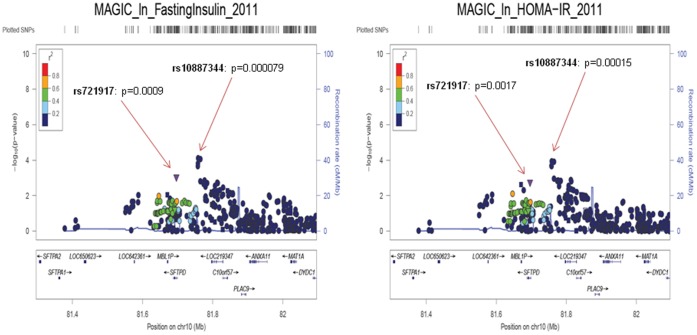
Regional association plot of chr.10. Regional association plot of the chromosome 10 region for fasting insulin and HOMA-IR in MAGIC GWA.

In order to validate the association between *SP-D* gene variants and T2D we explored the data generated in genome wide association (GWA) databases from the Meta-Analyses of Glucose and Insulin-related traits Consortium (MAGIC), who measures continuous glycaemic traits [24]. In this larger cohort, fasting Insulin (p = 0.0009) and HOMA_IR_ (p = 0.0017) were associated to rs721917 *SP-D* SNP following the additive model (***Figure 2***). Contrary to what might be expected, no significant changes regarding glucose concentrations were found.

**Figure 3:**
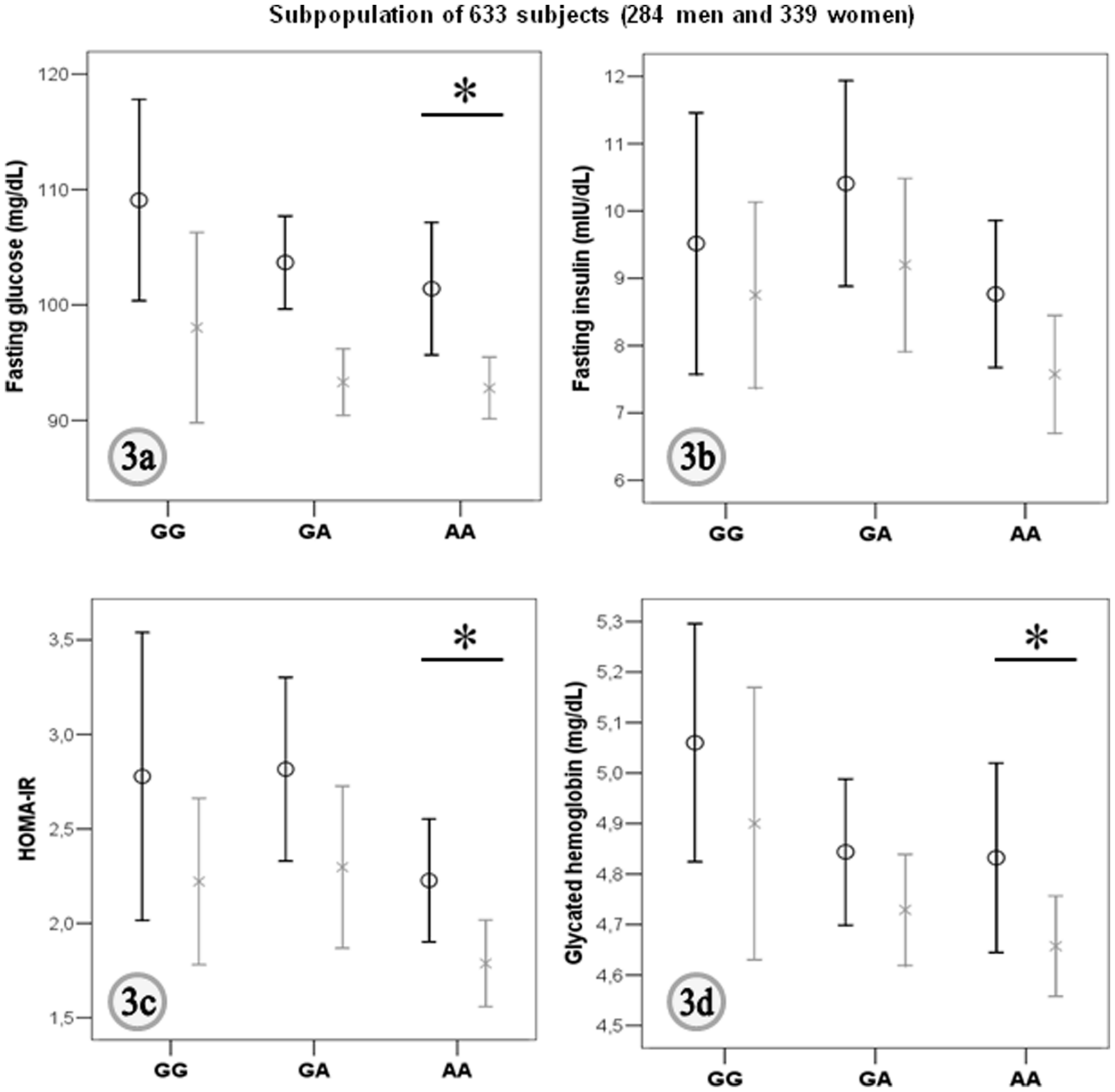
Genotypes for rs10887344 and measures of impaired glucose tolerance. Mean and 95% confidence interval for fasting glucose (***Fig. 3a***), fasting insulin (***Fig. 3b***), HOMA-IR (***Fig. 3c***), and glycated hemoglobin (***Fig. 3d***) according to the genotypes for rs10887344. Men: black bars, women: grey bars. *p<0.05 for comparisons with GG-genotype.

In addition to the association of the *SP-D* gene polymorphism rs721917, a second SNP (rs10887344; chr10∶81768162), located between *SP-D* gene and *LOC219347*, 59.3 Kb upstream of the *SP-D* gene (but not in LD with the first polymorphism analyzed, the rs721917, r^2^ = 0.082), was also associated with fasting insulin (p = 0.000078; ***Figure 2***) and HOMA_IR_ in this GWA. Of note, associations of this intergenic SNP (rs10887344) with parameters of insulin resistance were replicated in our sample (***Table 4***). Indeed, in agreement with results from MAGIC GWA, increased fasting glucose (p = 0.04; ***Fig. 3a***), fasting insulin (***Fig. 3b***), HOMA_IR_ (p = 0.022; ***Fig. 3c***), and glycated hemoglobin (p = 0.015; ***Fig. 3d***) was found in GG carriers for rs10887344 when compared to AA genotype (***Table 4***), independently of sex, BMI, and circulating SP-D. Then, according to results for this second SNP associated with *SP-D* gene (rs10887344), GG homozygotes had higher prevalence of T2D than A-allele carriers.

**Table 4:** Subjects’ characteristics according to the intergenic single nucleotide polymorphism rs10887344 (NC_000010.10:g.81768162G >A).

Genotype	GG	GA	AA	p*	p†
**Subjects** **(M/F)**	65/82	135/145	84/112		
**Smokers (%)**	20	29	24		
**T2D (%)**	**14**	**8**	**8**		
**Age (years)**	52±13	53±12	51±13	0.139	0.251
**BMI (kg/m^2^)**	28.2±4.7	28.2±4.8	27.6±4.5	0.295	0.227
**WHR**	0.88±0.07	0.89±0.09	0.87±0.09	0.054	0.382
**Fasting Glucose** **(mg/dL)**	**102.9±36.8**	**98.3±21.3**	**96.5±20.8**	0.065	**0.041**
**Glucose 2** **h-post** **overload (mg/dL)**	117.1±56.7	116.8±50.3	108.4±43.2	0.166	0.128
**Fasting insulin** **(mUI/dL)**	**9.1±7**	**9.8±8.4**	**8.1±4.8**	**0.039**	**0.118**
**HOMA_IR_**	**2.5±2.5**	**2.5±2.7**	**2.1±1.4**	**0.026**	**0.022**
**HbA_1c_ (%)**	**5.0±1.1**	**4.8±0.8**	**4.7±0.7**	**0.026**	**0.015**
**SP-D** **(ng/mL)**	97.2±53.6	103.8±63.8	98.3±55.5	0.524	0.864

**T2D:** Type 2 diabetes; **BMI:** Body mass index; **WHR:** Waist-to-hip ratio; **HbA_1c_:** Glycated hemoglobin; **SP-D:** Surfactant protein-D; **MBL:** Mannose binding lectin.

* for the comparison by ANOVA among the different genotypes, and

† for the comparison by test *t*-student between AA and GG carriers in the whole cohort. Significant differences are shown in **bold**.

## Discussion

Polymorphisms of common receptors in the innate immune system like mannose-binding lectin [29], and toll-like receptors [30] are associated with altered susceptibility to infection, and the risk of a variety of inflammatory diseases. Indeed, many genetic variants in the innate immune factors are associated with several metabolic risk factors for T2D, an observation that provides a rationale for further studying their role as biomarkers for the early risk prediction of this disease [31], [32].

Genetic variations in the coding region of *surfactant protein-D* (*SP-D*) are associated with an increased risk of T2D (current report). Surfactant collectins such as SP-D are well known to be involved in lung innate immunity, being a key factor in the prevention of respiratory infections (see [33] for a review). In a scenario of repeated injuries and exposure to infectious agents, a chronic inflammatory response has been proposed as a leading factor for the development of metabolic disturbances and insulin resistance [34]. Lipopolysaccharides (LPS), for example, are at the onset of high-fat diet-induced metabolic diseases by triggering low grade chronic inflammation [35].

Surfactant immune function is primarily attributed to SP-A and -D. These proteins opsonize pathogens, regulate the inflammatory response, and interact with the adaptive immune response, being involved in the susceptibility to inflammation and infection [36]. In a previous manuscript, we found cross-sectional and longitudinal associations of circulating SP-D concentrations with insulin resistance and T2D [19]. In this study, we describe that the 31 Met→Thr *SP-D* gene single nucleotide polymorphism (SNP) rs721917 was associated with insulin resistance and the prevalence of T2D. Interestingly, differences in fasting glucose between groups after controlling for origin, age, and gender remained significant when controlling for SP-D concentrations in plasma. This suggests that the possible influence of the SNP rs721917 on metabolism may arise from changes in the molecular structure and/or functional properties of SP-D protein rather than through changes in its circulating concentrations. Indeed, this non-synonymous polymorphic variation of the N-terminal domain of the SP-D affects oligomerization and function, as well as circulating concentrations [10], [11], changes that may explain the relationship with insulin resistance and T2D. In fact, subjects carrying the AA genotype (Met/Met) showed increased circulating SP-D concentrations in parallel to increased fasting glucose and T2D prevalence, especially among women and independently of circulating SP-D.

The putative functional consequences of rs721917 were explored using the FastSNP SNP characterization online application (http://fastsnp.ibms.sinica. *edu.tw/*). The SNP rs721917 involves a *missense* variation leading to conservative changes that appeared not only to affect the amino acid sequence of the protein but also to cause a disruption of three Exonic Splicing Enhancer (ESE) sequences, according to the systematic analysis of ESE sequences by Fairbrother *et al.* [37]. As three ESE are disrupted by this SNP (***Figure S1***), it is very likely that this SNP has an effect on alternative splicing, altering stability and circulating concentrations and even affecting biological functions. Accordingly, Leth-Larsen *et al.* [11] demonstrated that GG-carriers (Thr/Thr) produce almost exclusively monomers of SP-D, whereas subjects carrying AA genotype show multimers such as dodecamers and trimers of subunits. These high weight polimers of SP-D may bind to viruses and both Gram-positive and negative bacteria, while the monomeric species seem to bind LPS almost exclusively [11]. Thereby, the different genotypes may predispose individuals to different buffering capacity of metabolic endotoxemia. Indeed, the codons corresponding to this amino acid residue in the mature protein, here found to be linked to T2D, were associated with increase susceptibility to immune-related diseases [38], [39]. Interestingly, in a subpopulation of 71 subjects (25 AA, 31 AG, and 15 GG-subjects), we observed an inverse association between free LPS concentrations in plasma and circulating SP-D in GG subjects (r = **−**0.50, p = 0.05), but not among A allele carriers (data not shown).

On the other hand, our results indicated that gender may influence the degree of susceptibility to T2D of subjects who share the AA genotype. It is becoming increasingly recognized that there are gender differences in pulmonary disease susceptibility and severity, being women apparently more sensitive to pulmonary injury than men [40], [41]. Indeed, there are well-known gender-related differences in the composition of surfactant lipids and lipoproteins such as the surfactant protein B and C [42], [43]. Our results indicate that the presence of the variant *SP-D* allele (Met/Met) increases the odds of developing insulin resistance and T2D preferentially among women.

The analyses of SP-D variants in GWAS datasets from MAGIC reinforce the association with T2D. In these datasets, the associations of insulin resistance with this SNP, but also with different SNPs other than those genotyped for this study, suggest that the influence of rs721917 variant might be capturing the association of other variants, such as rs10887344. Concurrently, the effects of this variant on circulating SP-D and insulin sensitivity were tested in a subpopulation of this study confirming the association with the prevalence of T2D in both men and women, although not changing plasma SP-D concentrations. Then, the rs10887344 turns to be a promising variant to test in further large scale association studies. Unfortunately, we were not able to test this SNP in other large published case-control data, as this SNP was not directly typed by any of these studies and genotype data at the individual level was not available in order to impute it from the datasets.

Overall, the findings reported here suggest the implication of SP-D variants in the modulation of metabolic parameters. The *SP-D* gene polymorphism rs721917, beyond modifying circulating concentrations, may modify SP-D structure, changing its specificity against infectious agents and other external agents [11]. Triggering factors such as weight gain, aging, and repeated usual-life infections, among others, results in low-grade chronic inflammation which would be amplified in A-allele carriers. In the long term this process worsens insulin resistance, leading to T2D. However, the mechanisms responsible for the different associations should be investigated further.

## Supporting Information

Figure S1 **Functional aspects of rs721917.** Functional aspects of rs721917 according to the *FastSNP* SNP characterization online application (http://fastsnp.ibms.sinica. edu.tw/).
(TIF)
